# Strategy for the Enzymatic Acylation of the Apple Flavonoid Phloretin Based on Prior α-Glucosylation

**DOI:** 10.1021/acs.jafc.3c09261

**Published:** 2024-02-13

**Authors:** Jose L. Gonzalez-Alfonso, Cristina Alonso, Ana Poveda, Zorica Ubiparip, Antonio O. Ballesteros, Tom Desmet, Jesús Jiménez-Barbero, Luisa Coderch, Francisco J. Plou

**Affiliations:** † Institute of Catalysis and Petrochemistry (ICP-CSIC), Marie Curie 2, 28049 Madrid, Spain; ‡ Institute of Advanced Chemistry of Catalonia (IQAC-CSIC), Jordi Girona 18–26, 08034 Barcelona, Spain; § CIC bioGUNE, Basque Research and Technology Alliance (BRTA), 48160 Derio, Spain; ∥ Centre for Synthetic Biology (CSB), Ghent University, Coupure Links 653, 9000 Ghent, Belgium; ⊥ Basque Foundation for Science, 48009 Bilbao, Spain

**Keywords:** flavonoids, dihydrochalcones, antioxidants, acylation, hydrophile–lipophile balance (HLB)

## Abstract

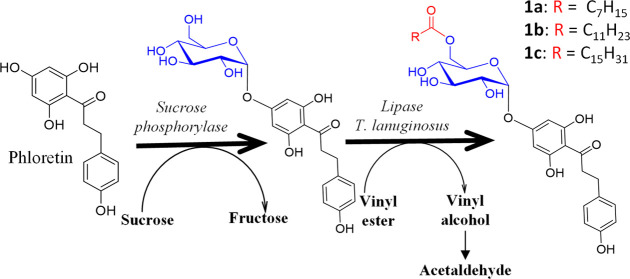

The acylation of flavonoids serves as a means to alter their physicochemical properties, enhance their stability, and improve their bioactivity. Compared with natural flavonoid glycosides, the acylation of nonglycosylated flavonoids presents greater challenges since they contain fewer reactive sites. In this work, we propose an efficient strategy to solve this problem based on a first α-glucosylation step catalyzed by a sucrose phosphorylase, followed by acylation using a lipase. The method was applied to phloretin, a bioactive dihydrochalcone mainly present in apples. Phloretin underwent initial glucosylation at the 4′-OH position, followed by subsequent (and quantitative) acylation with C8, C12, and C16 acyl chains employing an immobilized lipase from *Thermomyces lanuginosus*. Electrospray ionization-mass spectrometry (ESI-MS) and two-dimensional nuclear magnetic resonance spectroscopy (2D-NMR) confirmed that the acylation took place at 6-OH of glucose. The water solubility of C8 acyl glucoside closely resembled that of aglycone, but for C12 and C16 derivatives, it was approximately 3 times lower. Compared with phloretin, the radical scavenging capacity of the new derivatives slightly decreased with 2,2-diphenyl-1-picrylhydrazyl (DPPH) and was similar to 2,2-azino-bis(3-ethylbenzothiazoline-6-sulfonic acid) (ABTS^•+^). Interestingly, C12 acyl-α-glucoside displayed an enhanced (3-fold) transdermal absorption (using pig skin biopsies) compared to phloretin and its α-glucoside.

## Introduction

Polyphenols are a class of natural compounds widely found in fruits, vegetables, and other plant-based sources. Flavonoids are a specific subgroup of polyphenols characterized by a 15-carbon backbone consisting of two phenyl rings (A and B) connected by a heterocyclic ring (C) and encompass various subclasses such as flavones, flavonols, flavanones, isoflavones, anthocyanins, and others.^1^ Other polyphenols, such as phenolic acids and stilbenes, have distinct structures. Polyphenols possess a variety of biological activities and have garnered significant attention from the nutraceutical, pharmaceutical, and cosmetic industries.^2^ Their role involves safeguarding cells from the damaging effects of reactive oxygen species (ROS) and other free radicals.^3^ This explains their potential to prevent human diseases related to oxidative processes and cell damage, such as cancer, neurodegeneration, inflammatory disorders, diabetes, rheumatoid, or arthritis.^4−6^

The lipophilicity of a polyphenol is a crucial factor in its ability to traverse biological barriers such as cell membranes, the blood–brain barrier, and the skin, which are primarily composed of lipids.^7^ Optimizing the balance between lipophilicity and hydrophilicity is essential for the development of effective nutraceuticals with improved bioavailability and tissue penetration properties.^8^ The acylation of flavonoids also modifies their physicochemical properties, stability, and bioactivity.^9^ In this context, synthesized acylated derivatives of resveratrol have been assayed as antioxidants in several fish lipid matrices^10^ and also as cell-growth inhibitors of cancer prostate cells.^11^ González et al. demonstrated that alkyl gallates with medium-size chains (C6–C12) exhibited improved antioxidant activity in fish oil-in-water emulsions.^12^ However, excessive lipophilicity can have negative consequences, as highly lipophilic compounds may exhibit poor aqueous solubility, resulting in reduced dissolution and absorption rates.

The acylation of polyphenols using enzymes presents numerous advantages compared to chemical processes, including milder reaction conditions, enhanced specificity, sustainability, efficiency, compatibility with biological systems, and reduced waste and environmental impact.^13^ Many polyphenols commonly exist in the form of glycosides.^14^ The acylation of these molecules with different fatty and aromatic acids has been widely documented.^15−17^ In contrast, the acylation of nonglycosylated polyphenols proves to be more difficult since they have fewer available reactive sites for acylation, as they lack the sugar moiety that can act as a nucleophile.^18^ This limits the options for direct acylation and makes the reactions more challenging. Additionally, the presence of multiple phenolic groups in polyphenols can lead to side reactions or the need for protection and deprotection steps, further complicating the acylation process.^19^

We previously reported the acylation of resveratrol catalyzed by several lipases, but the reactions were quite slow when increasing the chain length of the fatty acid (75% conversion yield in 12 h for acetate, 55% in 160 h for stearate).^20^ This is a common pattern described in enzyme-catalyzed acylation processes over different nonglycosylated polyphenols.^21−25^ The only efficient cases of acylation of nonglycosylated polyphenols with long fatty acids occur when primary alcohols are present, which are present in certain compounds such as hydroxytyrosol^26,27^ or dihydromyrecitin.^28^ In other cases, the yields and/or regioselectivity often fail to meet satisfactory levels.^29^

In the present work, we propose a general two-step strategy for acylation of polyphenols that is based on a first α-glucosylation (catalyzed by a sucrose phosphorylase mutant) followed by acylation of the sugar moiety with vinyl esters using immobilized lipases.^30^ The R134A mutant of sucrose 6′-phosphate phosphorylase from *Thermoanaerobacterium thermosaccharolyticum* exhibits a significantly higher affinity for polyphenols than the native enzyme, caused by the increased size of the catalytic pocket.^31^ In particular, it proved to be successful for the glucosylation of a variety of polyphenols such as pyrogallol, alkyl gallates, resveratrol, quercetin, and different catechins.^32^ Recently, we reported the efficient synthesis of phloretin mono- and di-α-glucosides with this enzyme.^33^ For the acylation step, the lipase from *Thermomyces lanuginosus* has shown efficacy for glucose transesterification at the 6-OH position.^34^

As a proof of concept, we have applied this strategy to phloretin, a flavonoid belonging to the subgroup of dihydrochalcones, which is present in most parts of the apple tree, including the leaves, skin, and pomace of apples.^35^ Despite its reported antidiabetic, anticancer, and antiviral properties,^36,37^ its bioavailability is low as a consequence of its poor absorption.^38^ Due to the interest of phloretin in cosmetic applications,^39,40^ we further analyzed the percutaneous absorption of the synthesized acyl-α-glucosides and compared the results with the aglycone and the α-glucosides.

## Materials and Methods

### Enzyme and Reagents

The production of the recombinant sucrose phosphorylase mutant TtSPP_R134A from *T. thermosaccharolyticum* was carried out as previously described.^33^ The lipase from *T. lanuginosus* immobilized on granulated silica (Lipozyme TL IM, 250 IUN/g) was kindly provided by Novozymes. Phloretin was purchased from Hunan MT Health Inc. (Hunan, China). Sucrose was obtained from Scharlau. Vinyl octanoate and vinyl palmitate were obtained from TCI Chemicals. Vinyl laurate, ABTS [2,2-azino-bis(3-ethylbenzothiazoline-6-sulfonic acid)], DPPH (2,2-diphenyl-1-picrylhydrazyl), and (R)-Trolox (6-hydroxy-2,5,7,8-tetramethylchroman-2-carboxylic acid) were acquired from Sigma-Aldrich. All other reagents and solvents were of the highest purity grade available.

### General Procedure for the Enzymatic Acylation of Phloretin

Phloretin was α-glucosylated with the R134A sucrose phosphorylase mutant from *T. thermosaccharolyticum* as described in a previous work.^33^ Phloretin 4′-*O*-α-d-glucopyranoside (7 mg, 16 μmol), vinyl ester (320 μmol), and Lipozyme TL IM (7 mg) were mixed in tert-butyl alcohol (1 mL). The reaction was carried out at 60 °C under vigorous shaking. Aliquots of 50 μL were taken at different times and diluted with 450 μL of methanol. Samples were analyzed by TLC and HPLC. TLC analysis was carried out with silica gel plates 60 F254 (Merck) with a mixture of ethyl acetate, methanol, and water 60:5:4 (v/v/v) as the mobile phase. The spots were visualized by UV light and also employing 10% (v/v) H_2_SO_4_ solution and heating the plate. HPLC analysis was performed using a quaternary pump (model 600, Waters) coupled to an autosampler (model ProStar 420, Varian Inc.). The column was a Zorbax Eclipse Plus C18 (4.6 mm × 100 mm, 3.5 μm, Agilent) at 40 °C. The detector was a photodiode array (ProStar, Varian), and peaks were detected at 297 nm and analyzed with the software Varian Star LC workstation 6.41. The mobile phase was acetonitrile and water in gradient, both solvents acidified with 0.1% (v/v) formic acid. The gradient was formed by increasing the acetonitrile from 15 to 95% in 5 min, followed by an isocratic step of 10 min at 95%. After that, the column was equilibrated under initial conditions for 5 min before the next injection.

### Product Purification

The acylation reaction was scaled up to an initial amount of 100 mg of phloretin glucoside (total volume of 14.3 mL). After 24 h of reaction, the solvent was evaporated with an R-210 rotary evaporator (Buchi), and the pellet was washed with toluene and water to separate the residual fatty acid and phloretin monoglucoside, respectively. After that, the solid was redissolved in methanol. The main product was purified by flash chromatography (Pure C-815 system, Buchi). A FlashPure EcoFlex cartridge (Buchi) with 12 g of silica (particle size, 50 μm) was used. The mobile phase consisted of a gradient with ethyl acetate and aqueous methanol at 60% (v/v) with a flow rate of 30 mL/min. The method started with 100% (v/v) of ethyl acetate during 2.6 min, followed by a gradient with aqueous methanol from 0% (v/v) to 15% (v/v) in 2.6 min. This phase was maintained for 2 min. The elution of products was detected with a photodiode array detector in series with an evaporative light-scattering detector (ELSD). Finally, the solvents were evaporated to obtain the corresponding acylated phloretin 4′-*O*-α-d-glucopyranoside. After purification, the products were collected as yellow oils and characterized by MS and 2D-NMR.

### Mass Spectrometry

The molecular weight of the main products was determined by high-resolution mass spectrometry with electrospray ionization (ESI) coupled to a hybrid QTOF analyzer (model MAXIS II, Bruker) in the positive reflector mode. Methanol with 0.1% formic acid was employed as the ionizing phase.

### Nuclear Magnetic Resonance (NMR) Analysis

The structure of the products was determined using a combination of 1D and 2D (^13^C-APT, COSY, DEPT-HSQC, and TOCSY) standard NMR techniques. The spectra of the samples, dissolved in CD_3_OD (ca. 7–13 mM), were recorded on a Bruker AV-III 600 spectrometer equipped with a PA TXI probe with gradients in the *X*, *Y*, and *Z* axis, at a temperature of 298 K. Chemical shifts were expressed in parts per million (ppm). Residual MeOD-*d*_4_ signal was used as an internal reference (3.31 ppm). All of the pulse sequences were provided by Bruker. For the DEPT-HSQC experiment, values of 7 ppm and 1K points for the ^1^H dimension and 165 ppm and 256 points for the ^13^C dimension were used. For the homonuclear COSY, 7 ppm windows were used with a 2K × 256 point matrix. For the HMBC experiment, values of 7 ppm and 2K points for the ^1^H dimension and 220 ppm and 384 points for the ^13^C dimension were used.

### Aqueous Solubility

The compounds were incubated at 25 °C in water (saturated conditions) during 3 days under orbital stirring (1000 rpm). Then, the samples were centrifuged, and the supernatants (200 μL) were analyzed in triplicate in a 96-well plate, using a phloretin calibration curve in methanol (0–200 μg/mL). The absorbance was measured at 297 nm in a UV–vis spectrophotometer (Tecan Infinite M200).

### Antioxidant Activity

The capacity of the synthesized derivatives to reduce the radical cation 2,2-azino-bis(3-ethylbenzothiazoline-6-sulfonic acid) diammonium salt (ABTS^•+^) and 2,2-diphenyl-1-picrylhydrazyl (DPPH) was assessed in 96-well plates.^33,41^ (R)-Trolox was employed as a reference in both assays. Standard solutions with concentrations between 0 and 200 μM for (R)-Trolox and between 0 and 1000 μM for phloretin and its acyl-glucosides were prepared in methanol. The compounds (20 μL) at different concentrations were mixed with 230 μL of ABTS^•+^ (previously diluted with methanol to get an absorbance of 0.7 at 655 nm) or 200 μL of DPPH (200 μM in methanol). After incubation in the dark at room temperature for 15 min, the absorbance (at 655 nm for ABTS^•+^ and at 540 nm for DPPH) was measured with a Zenyth 200 spectrophotometer. The EC_50_ was referred to the concentration of compound needed to reduce the ABTS^•+^ absorbance to 50%. The results were expressed as Trolox equivalent antioxidant capacity (TEAC), calculated from the EC_50_ of each compound and the EC_50_ of Trolox.

### Percutaneous Absorption

The study was carried out *in vitro* with pig biopsies placed on Franz static diffusion cells (3 mL, 1.86 cm^2^ of exposed area, diameter: 30 mm, Lara-Spiral, Courtenon, France) in order to determine the distribution of the active compound (API) at the different skin layers after an exposure time of 24 h. All materials and procedures for this test have been described in a previous report employing phloretin and two glucosides.^33^ The concentration of the extracted active compound was determined with the HPLC method described before.

## Results and Discussion

### Strategy for the Acylation of Phloretin

**Figure 1:**
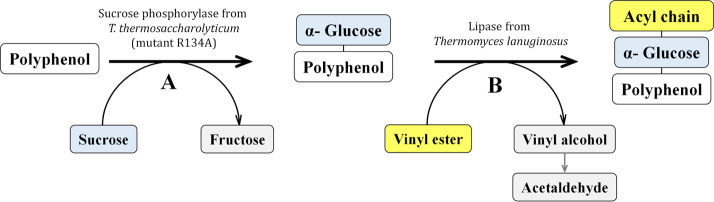
Reaction pathway proposed to obtain acylated derivatives of polyphenols. (A) The polyphenol is α-glucosylated using sucrose as the glucosyl donor catalyzed by the sucrose phosphorylase mutant R134A from *T. thermosaccharolyticum*. (B) The α-glucoside is acylated with vinyl esters (C8–C16) catalyzed by the lipase from *T. lanuginosus*, with high regioselectivity at 6-OH of the glucose, yielding the corresponding acyl α-glucosides.

The overall reaction pathway for the acylation process is illustrated in Figure 1. In the case of phloretin, α-glucosylation was carried out using 10% (v/v) acetone as the cosolvent to increase the solubility of the flavonoid.^33^ The selective formation of a monoglucoside or a diglucoside (with α(1 → 3) linkage between the two glucoses) can be kinetically controlled. The monoglucoside concentration reaches its maximum after approximately 12 h, with the remaining phloretin being almost negligible at that point, and subsequently, it decreases over time as the diglucoside emerges. The maximum conversion yield of the monoglucoside (phloretin 4′-*O*-α-d-glucopyranoside) was 53%, and this product showed a 71-fold greater aqueous solubility than aglycone.^33^

For the second step, *T. lanuginosus* lipase was selected due to its remarkable regioselectivity for the 6-OH of glucose in different acylation reactions.^42,43^*Tert*-butanol has proven to be an excellent solvent for the reactions catalyzed by this enzyme, offering noteworthy stability and activity for the biocatalyst.^44^ This solvent also provides remarkable solubility for the phloretin monoglucoside. Vinyl esters were chosen as acyl donors because the rate of transesterification of carbohydrates is about 20–100 times faster than with alkyl esters.^45,46^ Specifically, we explored the acylation of the phloretin α-glucoside employing vinyl esters with different fatty acid chains (C8, C12, and C16).

**Figure 2:**
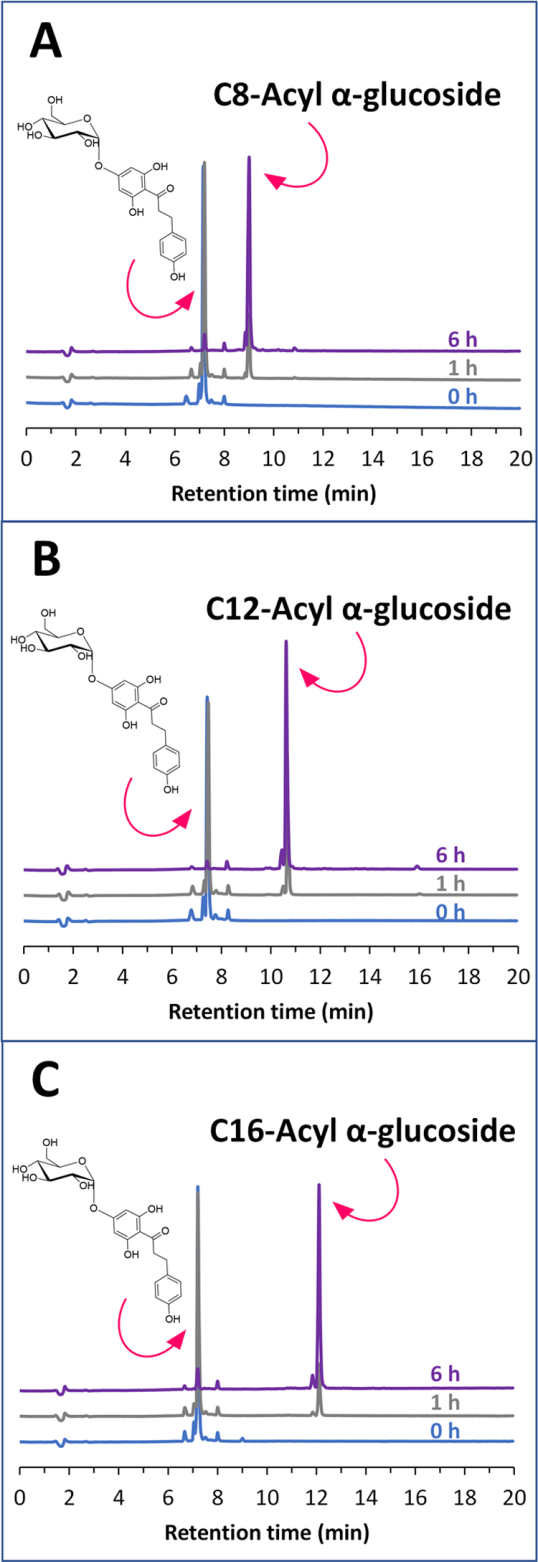
HPLC chromatograms (at 0, 1, and 6 h) showing the acylation of phloretin α-glucoside. Reaction conditions: phloretin 4′-*O*-α-glucopyranoside (0.016 mmol), vinyl ester (0.32 mmol), Lipozyme TL IM (7 mg), *tert*-butanol (1 mL), 60 °C. Acyl donors: (A) vinyl octanoate, (B) vinyl laurate, and (C) vinyl palmitate.

The reaction was carried out by mixing phloretin 4′-*O*-α-glucopyranoside (16 mM) with the vinyl ester (320 mM) in *tert*-butanol, in the presence of the immobilized lipase Lipozyme TL IM (7 mg/mL). The acylation reaction was maintained at 60 °C and monitored by TLC (a new spot with high *R*_f_ appeared in the three cases) and HPLC. The chromatograms at 0, 1, and 6 h are shown in Figure 2, where A is the reaction with vinyl octanoate, B with vinyl laurate, and C with vinyl palmitate. The appearance of a new peak (with a higher retention time than the glucoside) corresponding to the acylated α-glucoside of phloretin was evident in the three reactions.

**Figure 3:**
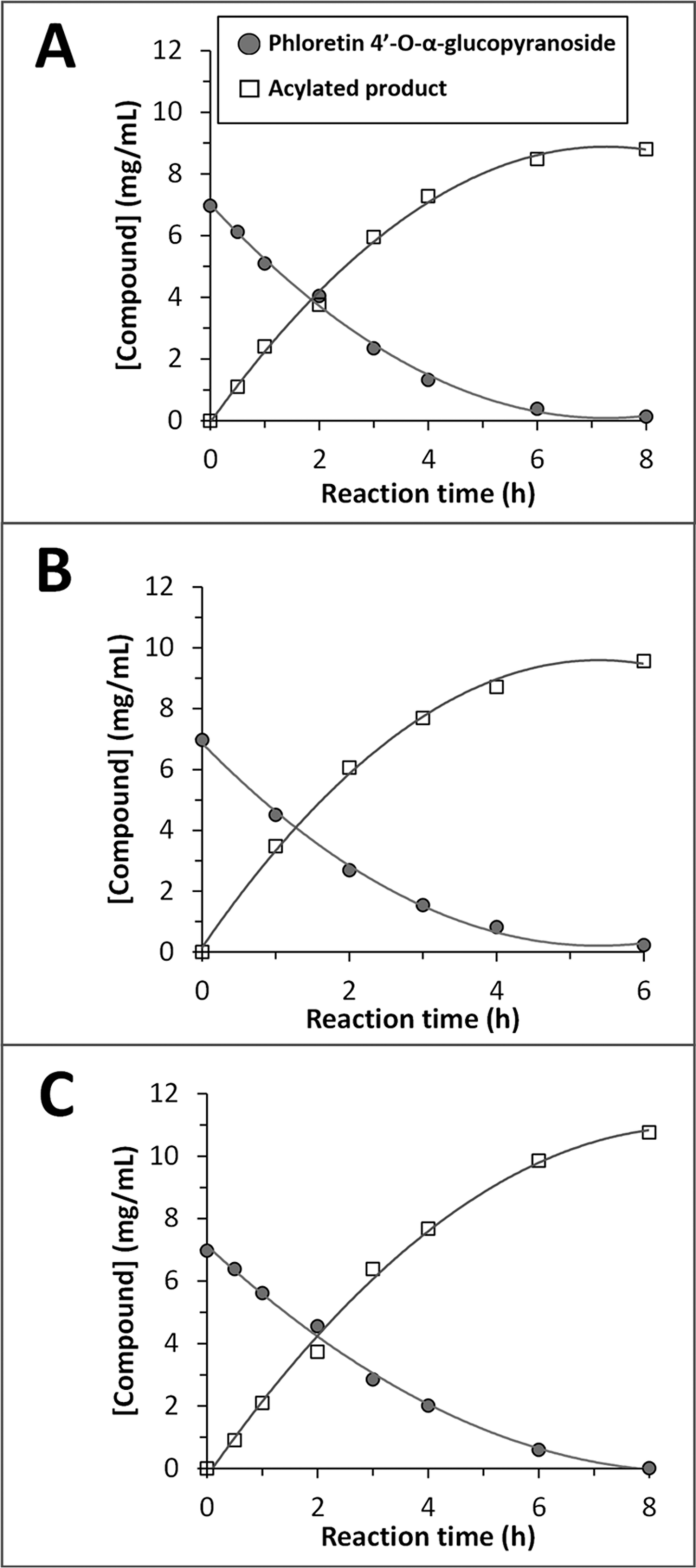
Progress of phloretin monoglucoside acylations. Reaction conditions: phloretin 4′-O-α-glucopyranoside (7 mg, 0.016 mmol), vinyl ester (0.32 mmol), Lipozyme TL IM (7 mg), *tert*-butanol (1 mL), 60 °C. Acyl donors: (A) vinyl octanoate, (B) vinyl laurate, and (C) vinyl palmitate.

The progress of the acylation reactions underwent further examination (Figure 3). The reactions were very fast and finished in 8 h for C8 and C16 and in 6 h for C12, reaching a conversion yield in all cases higher than 95%. The enzymatic reaction exhibited an exceptionally high level of efficiency, compared to nonglycosylated polyphenols, owing to the presence of the glucose moiety in the molecule. To illustrate this effect, Saik et al. synthesized quercetin oleate with the lipase B from *Candida antarctica*, and the conversion yield was approximately 25% in 7 days.^21^ Epigallocatechin gallate (EGCG) was acetylated by transesterification with vinyl acetate using immobilized lipase from *Mucor miehei* (83% conversion after 10 h), but there is no information regarding longer fatty acids.^47^ Recently, Cho et al. synthesized several acyl myricetins with *C. antarctica* B lipase, but the longest fatty acid was C8 and the reaction time was 96 h.^48^ Peng et al. reported a 4% yield of quercetin monolaurate using the lipase from *Burkholderia cepacia*(24) and a 9–15% yield with resveratrol.^25^

### Chemical Characterization of the Acylated Derivatives

The acylated derivatives were purified by flash chromatography (Figure S1) and characterized by exact mass spectrometry (ESI-MS, Figures S2–S4) and nuclear magnetic resonance (NMR, Figures S5–S11). Table 1 presents an overview of the NMR data.

**Table 1:** NMR Spectroscopy Data (600 MHz, DMSO-*d*_6_) for the Synthesized Compounds

	δ_C_ (ppm), type			δ_H_ (ppm), *J* in Hz		
position	**1a**	**1b**	**1c**	**1a**	**1b**	**1c**
1	134.0, C	134.0, C	133.9, C			
2/6	130.5, CH	130.5, CH	130.5, CH	7.04 (app d, *J* = 8.5, 2H)^a^	7.0 (app d, *J* = 8.5, 2H)^a^	7.04 (app d, *J* = 8.5, 2H)^a^
3/5	116.3, CH	116.3, CH	116.3, CH	6.69 (app d, *J* = 8.5, 2H)^a^	6.7 (app d, *J* = 8.5, 2H)^a^	6.69 (app d, *J* = 8.5, 2H)^a^
4	156.7, C	156.6, C	156.7, C			
7	31.4, CH_2_	31.4, CH_2_	31.4, CH_2_	2.86 (m, 2H),	2.86 (m, 2H)	2.86 (m, 2H)
8	47.8, CH_2_	47.8, CH_2_	47.8, CH_2_	3.31 (m, 2H)	3.3 (m, 2H)	3.31 (m, 2H)
9	207.2 C	207.2, C	207.1, C			
1’	107.1, C	107.1, C	107.1, C			
2′/6′	165.5, C	165.4, C	165.5, C			
3′/5′	96.9, CH	96.9, CH	96.9, CH	6.17 (s, 2H)	6.20 (s, 2H)	6.16 (s, 2H)
4′	164.4, C	164.3, C	164.4, C			
1″	98.1, CH	98.0, CH	98.1, CH	5.51 (d, *J* = 3.7, 1H)	5.5 (d, *J* = 3.7, 1H)	5.51 (d, *J* = 3.7, 1H)
2″	73.1, CH	73.1, CH	73.1, CH	3.58 (dd, *J* = 3.7, 9.7. 1H),	3.6 (dd, *J* = 3.7, 9.7, 1H)	3.57 (dd, *J* = 3.7, 9.7, 1H)
3″	75.0, CH	75.0, CH	75.0, CH	3.78 (dd, *J* = 8.8, 9.7, 1H)	3.8 (dd, *J* = 9.3, 9.7, 1H),	3.78 (dd, *J* = 9.3, 9.7, 1H)
4″	72.1, CH	72.1, CH	72.1, CH	3.30 (dd, *J* = 8.8, 9.8, 1H)	3.3 (dd, *J* = 9.3, 10.0, 1H)	3.29 (dd, *J* = 9.3, 10.0, 1H)
5″	72.5, CH	72.5, CH	72.5, CH	3.73 (ddd, *J* = 2.1, 7.4, 9.8, 1H)	3.7 (ddd, *J =* 2.1, 7.8, 10.0, 1H)	3.74 (ddd, *J =* 2.0, 7.7, 10.0, 1H)
6″	64.7, CH_2_	64.8, CH_2_	64.8, CH_2_	4.11 (dd, *J* = 7.4, 11.7, 1H, H6″a) 4.42 (dd, *J* = 2.0, 11.8, 1H, H6″b)	4.1 (dd, *J* = 7.6, 11.7, 1H, H6″a) 4.4 (dd, *J* = 7.4, 1.9, 1H, H6″b)	4.1 (dd, *J* = 7.6, 11.8, 1H, H6″a) 4.42 (1H, *J* = 2.0, 11.8, 1H H6″b)
1a	175.5, C	175.6, C	175.5, C			
2a	35.1, CH_2_	35.2, CH_2_	35.2, CH_2_	2.23 (m, 2H)	2.2 (m, 2H)	2.22 (m, 2H)
3a	26.0, CH_2_	26.0, CH_2_	26.1, CH_2_	1.47 (m, 2H)	1.46 (m, 2H)	1.45 (m, 2H)
4a	30.3, 30.2, CH_2_^b^	30.4–31, CH_2_^b^	31.0–30.4, CH_2_^b^	1.20 (m, 4H)	1.17–1.35 (m, 12H)	1.22–1.27 (m, 20H)
5a						
6a	33.0, CH_2_			1.21 (m, 2H)		
7a	23.8, CH_2_			1.28 (m, 2H),		
8a	14.6, CH_3_			0.88 (t *J* = 7.3, 3H)		
9a						
10a		33.0, CH_2_			1.27 (m, 2H)	
11a		23.9, CH_2_			1.30 (m, 2H)	
12a		14.6, CH_3_			0.89 (t *J* = 7.0, 3H)	
13a						
14a			33.2, CH_2_			1.28 (m, 2H)
15a			23.9, CH_2_			1.31 (m, 2H)
16a			14.6, CH_3_			0.89 (t *J* = 7.1, 3H)

a Apparent doublet, it is the component of the second-order AAXX’ spin system expected for a 1,4-disubstituted phenyl ring.

b Not distinguishable due to overlapping.

**Figure 4:**
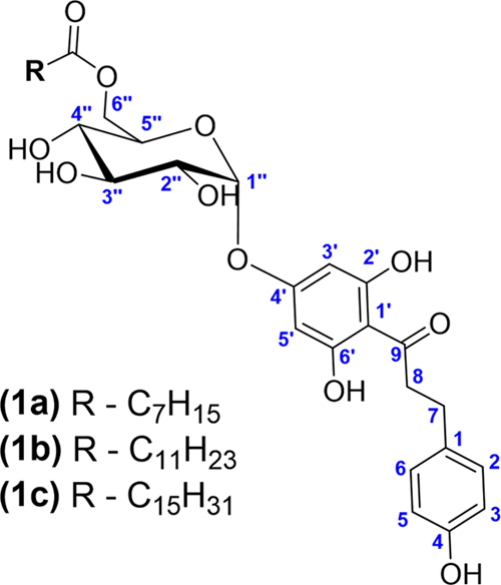
Molecular structure of the synthesized compounds: (**1a**) phloretin 4′-*O*-(6-*O*-octanoyl)-α-d-glucopyranoside; (**1b**) phloretin 4′-*O*-(6-*O*-lauroyl)-α-d-glucopyranoside; and (**1c**) phloretin 4′-*O*-(6-*O*-palmitoyl)-α-d-glucopyranoside.

The acylation position of the acylated derivatives was determined by NMR through the analysis of 2D-HMBC. The two protons at position 6″- of the glucose presented an HMBC signal with the first carbon of the alkyl chain, which allowed us to confirm the 6″-OH of glucose as the acylation point with high regioselectivity. Furthermore, HMBC signals were observed between the anomeric 1″H of glucose and the 4′-C of the phloretin, with a *J* of 3.7 Hz, indicating α configuration. To the best of our knowledge, the three synthesized acylated derivatives (Figure 4) are new compounds. The characterization data for each compound is described below.

#### Phloretin 4′-*O*-(6-*O*-Octanoyl)-α-d-glucopyranoside (**1a**)

Conversion yield: 98%; yield: 76%, 98 mg; yellow oil; HPLC-UV (297 nm): *t*_R_ 9.0 min (90% purity); ESI-MS (*m*/*z*): 585.2306 [M + Na]^+^, calculated 585.2312.

#### Phloretin 4′-*O*-(6-*O*-Lauroyl)-α-d-glucopyranoside (**1b**)

Conversion yield: 97%; yield: 73%, 103 mg; yellow oil; HPLC-UV (297 nm): *t*_R_ 10.6 min (89% purity); ESI-MS (*m*/*z*) 641.2925 [M + Na]^+^, calculated 641.2938.

#### Phloretin 4′-*O*-(6-*O*-Palmitoyl)-α-d-glucopyranoside (**1c**)

Conversion yield: >99%; yield: 71%, 100 mg; yellow oil; HPLC-UV (297 nm): *t*_R_ 12.4 min (97%); ESI-MS (*m*/*z*) 697.3564 [M + Na]^+^, calculated 697.3564.

### Aqueous Solubility and Stability

The solubility in water of the three acylated derivatives was analyzed at 25 °C and compared with the results previously obtained with phloretin monoglucoside and aglycone. The results are summarized in Table 2. As expected, the acylation of the monoglucoside decreased the water solubility. It is noteworthy that the effect of glucosylation, followed by acylation with C8, has a negligible effect on the initial solubility of phloretin. On the other hand, the solubility of the C12 and C16 derivatives was 3 times lower than that of aglycone. A similar increase in lipophilicity and the partition coefficient (log *P*) upon acylation has also been reported for other flavonoids.^49,50^

**Table 2:** Water Solubility at 25 °C of Phloretin, Phloretin α-Glucoside, and the New Acylated α-Glucosides

compound	solubility (mg/L)
phloretin^a^	23.2 ± 0.3
phloretin 4′-*O*-α-d-glucoside^a^	1644 ± 101
phloretin 4′-*O*-(6-*O*-octanoyl)-α-d-glucoside	22.1 ± 0.5
phloretin 4′-*O*-(6-*O*-lauroyl)-α-d-glucoside	6.0 ± 0.2
phloretin 4′-*O*-(6-*O*-palmitoyl)-α-d-glucoside	7.1 ± 0.3

a Data available in a previous article.^33^

Regarding the stability, we observed that the synthesized acyl-glucosides exhibit significant stability over several days under neutral and mildly acidic pH conditions (Figure S12). However, at pH 8.0, we noted a gradual hydrolysis of the ester bond, resulting in approximately 25% conversion to the respective monoglucoside over an 8-day period. The compounds were incubated in a 70:30 (v/v) mixture of ethanol and 100 mM buffer (sodium acetate for pH 4.0; sodium phosphate for pH 6.0 and 8.0), at 37 °C. In this context, Švehlíková et al. reported that acetyl and malonyl glucosides of the flavonoid apigenin were unstable depending on the storage conditions and the acylation position on the glucose moiety (the derivatives at the primary hydroxyl 6-OH were more stable in comparison with the secondary hydroxyl groups).^51^ In our work, the presence of a long fatty acid chain and the acylation at 6″-OH can promote significant stability of the synthesized derivatives.

### Antioxidant Properties

**Figure 5:**
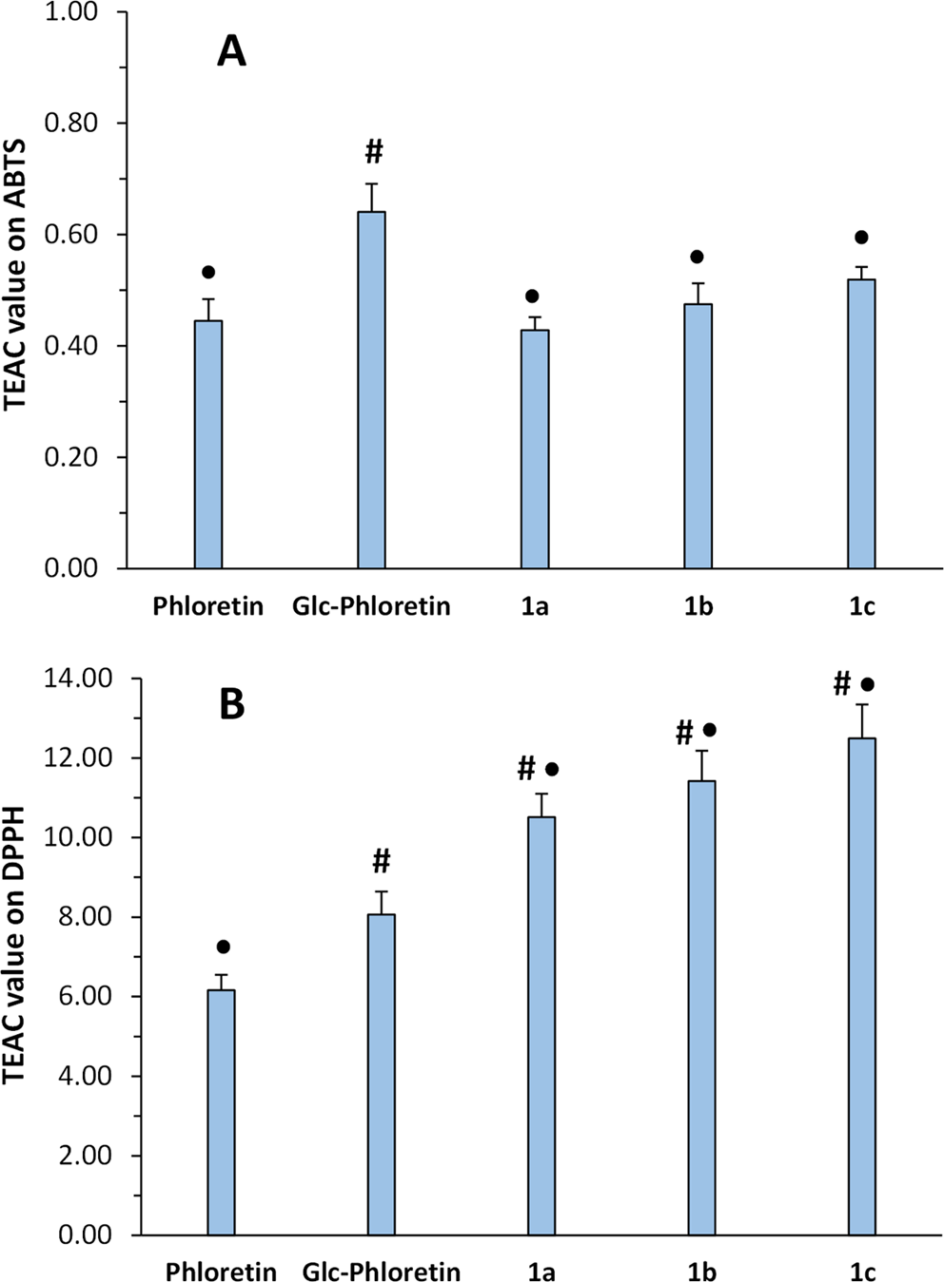
Antioxidant activity on ABTS^•+^ (A) and DPPH (B) of phloretin, its 4′-O-α-glucoside, and the corresponding acylated α-glucosides. Data are expressed as TEAC value ± SD (*n* = 3, ^#^*p* < 0.01 vs phloretin; ^●^*p* < 0.02 vs Glc-phloretin).

The antioxidant activity of the synthesized acylated derivatives was evaluated measuring the Trolox equivalent antioxidant capacity (TEAC), which assesses the ability of a compound to scavenge free radicals and prevent oxidative damage.^52^ First, the compounds were tested for their ability to neutralize the radical cation ABTS^•+^ (2,2’-azinobis-(3-ethylbenzothiazoline-6-sulfonic acid)). After the different compounds were added, the decrease in color intensity was measured spectrophotometrically. The assay was also performed with 1,1-diphenyl-2-picrylhydrazyl (DPPH), which relies on the ability of a substance to donate hydrogen atoms or electrons to neutralize the stable free radical DPPH. In both cases, the antioxidant capacity is typically expressed in terms of Trolox equivalents, a reference synthetic compound with known antioxidant activity. The TEAC values were calculated from EC_50_, i.e., the concentration of each compound needed to reduce the ABTS^•+^ or DPPH absorbance to 50%. The main results are presented in Figure 5.

The acylated derivatives exhibited a slightly higher antioxidant activity in the ABTS^•+^ assay than the corresponding α-glucoside and similar to the aglycone.^33^ The increase of antioxidant activity upon acylation has also been reported in other flavonoids such as proanthocyanidin^53^ and naringin.^54^ It is noteworthy that phloretin and its derivatives showed antioxidant activity higher than that of Trolox in this assay.

In contrast, the acylated derivatives displayed an antioxidant activity lower than those of phloretin and the α-glucoside in the DPPH assay. In this case, the antioxidant activity was significantly lower than that of Trolox. It is reported that the DPPH assay usually underestimates the dihydrochalcone antioxidant activity.^55^

### Skin Absorption Study

Enzymatic acylation can also be regarded as a strategy to increase the skin absorption of bioactive polyphenols. The capacity of phloretin to protect against UV radiation, its antimicrobial activity, and its antioxidant power offer a great potential for advanced applications in cosmetics.^56,57^ Phloretin is also employed as a penetrator enhancer for other bioactive substances.^56^ In a previous work, we reported the skin absorption of phloretin, along with its monoglucoside and diglucoside.^33^ Phloretin and the monoglucoside displayed a similar absorption pattern; however, the absorption of diglucoside was reduced.

**Figure 6:**
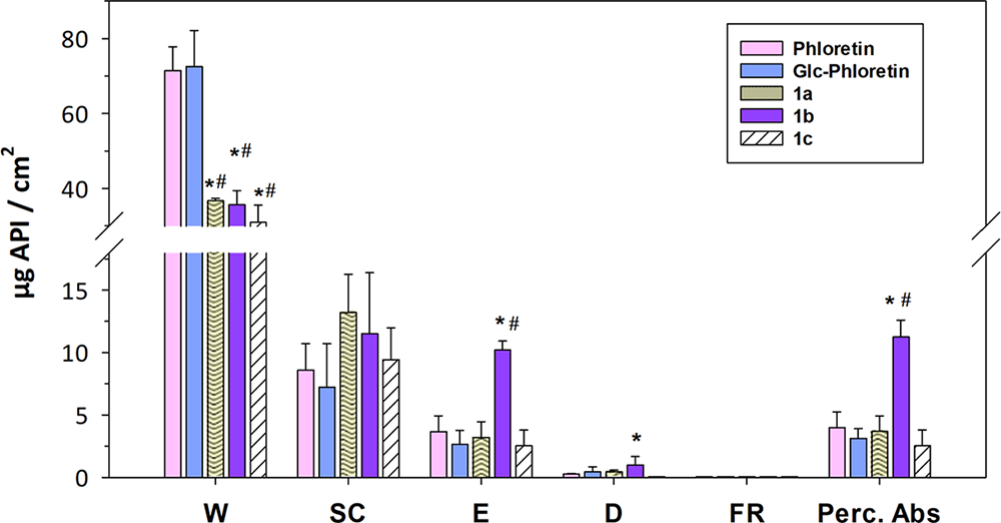
*In vitro* percutaneous absorption of acylated derivatives of phloretin and their precursors (phloretin and its 4′-*O*-α-glucoside) within the different skin layers. W is the surface excess, SC is the stratum corneum, E is the epidermis, D is the dermis, FR is the fluid receptor, and Perc. Abs. is the percutaneous absorption. The results are expressed as μg/cm^2^ of the active pharmaceutical ingredient (API). Mean values ± standard deviations, *n* = 3; *p < 0.05 vs phloretin; ^#^*p* < 0.05 vs α-Glc-phloretin.

When seeking to improve transdermal absorption and facilitate effective penetration through the skin barriers, simple glycosylation is typically not regarded as a favorable strategy. In contrast, the acylation of the glucoside could increase skin absorption. We measured the percutaneous absorption of the synthesized acyl-glucoside derivatives of phloretin using pig skin biopsies (Figure S13). The skin absorption of the aglycone (phloretin) and the 4′-*O*-α-d-glucopyranoside, reported in a previous work,^33^ is also shown to facilitate the comparison. Figure 6 illustrates the main results obtained within the different skin layers.

Remarkably, the lauroyl ester **1b** exhibited a greater percutaneous absorption (approximately 3-fold increase) than phloretin and 4′-*O*-α-glucoside. However, the acylation with C8 and C16 had no significant effect on skin absorption. The absorption through epithelium barriers depends on the hydrophile–lipophile balance (HLB) and the molecular size of the compounds.^58^ Within this context, Yang et al. reported that acylation of anthocyanins was related to a better interaction with functional proteins and membrane lipids.^59^

In summary, we developed a useful (and general) strategy for the synthesis of acylated derivatives of flavonoid aglycones. The method is based on a previous (and efficient) α-glucosylation; the glucosyl moiety provides an acylation site for regiospecific lipases. We applied this strategy to the flavonoid phloretin, which led to the synthesis of new acyl-glucosides with high conversion rates and outstanding regioselectivity. The C12-acylated derivative exhibited a superior skin penetration efficiency. The use of different acyl donors could provide a way to modulate the physicochemical properties of polyphenols as well as their biological properties in terms of *in vivo* absorption and bioavailability.

## Supporting Information Available

Figure S1, purification by flash chromatography of compound **1a**; Figures S2–S11, detailed MS and NMR spectra for the new compounds; Figure S12, stability of compounds **1a** and **1c** in a 70:30 (v/v) mixture of ethanol and 100 mM buffer, at 37 °C; and Figure S13, representation of *in vitro* percutaneous absorption experiments. (PDF)

## Abbreviations

ABTS: 2,2-azino-bis(3-ethylbenzothiazoline-6-sulfonic acid)
DPPH: 2,2-diphenyl-1-picrylhydrazyl
(R)-Trolox: 6-hydroxy-2,5,7,8-tetramethylchroman-2-carboxylic acid
TEAC: trolox equivalent antioxidant capacity
MS: mass spectrometry
ESI: electrospray ionization
NMR: nuclear magnetic resonance
^13^C-APT: attached proton test
COSY: correlation spectroscopy
HSQC: heteronuclear single quantum coherence
HMBC: heteronuclear multiple bond correlation
HLB: hydrophile–lipophile balance
