# Supplementary material for: Strategy for the Enzymatic Acylation of the Apple Flavonoid Phloretin Based on Prior α-Glucosylation
Source: J Agric Food Chem. 2024 Feb 13;72(8):4325–33. doi: 10.1021/acs.jafc.3c09261 (PMC10905995; doi:10.1021/acs.jafc.3c09261)
Supplement: Supplementary file 1 — jf3c09261_si_001.pdf [file jf3c09261_si_001.pdf]

Supporting Information

Strategy for the enzymatic acylation of the apple flavonoid phloretin based on prior α-glucosylation

Jose L. Gonzalez-Alfonso,[a] *Cristina Alonso,*[b] *Ana Poveda,*[c] *Zorica Ubiparip,*[d] *Antonio O. Ballesteros,*[a] *Tom Desmet,*[d] *Jesús Jiménez-Barbero,*[c,e] *Luisa Coderch,*[b] *and Francisco J. Plou* [a,*].

[a] Institute of Catalysis and Petrochemistry (ICP-CSIC), Marie Curie 2, 28049 Madrid, Spain.

[b] Institute of Advanced Chemistry of Catalonia (IQAC-CSIC), Jordi Girona 18-26, 08034 Barcelona, Spain.

[c] CIC bioGUNE, Basque Research and Technology Alliance (BRTA), 48160 Derio, Spain.

[d] Centre for Synthetic Biology (CSB), Ghent University, Coupure Links 653, 9000 Ghent, Belgium.

[e] Basque Foundation for Science, 48009 Bilbao, Spain.

**Figure S1**	Purification by flash chromatography of phloretin 4'-O-(6-O-octanoyl)-α-D-glucopyranoside (**1a**)
**Figure S2**	ESI-MS Phloretin 4'-O-(6-O-octanoyl)-α-D-glucopyranoside (**1a**)
**Figure S3**	ESI-MS Phloretin 4'-O-(6-O-lauroyl)-α-D-glucopyranoside (**1b**)
**Figure S4**	ESI-MS Phloretin 4'-O-(6-O-palmitoyl)-α-D-glucopyranoside (**1c**)
**Figure S5 (A)**	1H-NMR of **1a**

**Figure S5 (B)**	13C-NMR of **1a**
**Figure S6 (A)**	2D-HSQC of **1a**
**Figure S6 (B)**	2D-HMBC of **1a**
**Figure S7 (A)**	1H-NMR of **1b**
**Figure S7 (B)**	13C-NMR of **1b**
**Figure S8 (A)**	2D-HSQC of **1b**
**Figure S8 (B)**	2D-HMBC of **1b**
**Figure S9 (A)**	1H-NMR of **1c**
**Figure S9 (B)**	13C-NMR of **1c**
**Figure S10 (A)**	2D-HSQC of **1c**
**Figure S10 (B)**	2D-HMBC of **1c**
**Figure S11**	1H-NMR spectra of compounds **1a**, **1b** and **1c**. Some impurities are marked and identified as ethanol (*), methanol (◊) and free acid (○).
**Figure S12**	Stability of compounds **1a** and **1c** in a 70:30 (v/v) mixture of ethanol and 100 mM buffer, at 37 ˚C.
**Figure S13**	Scheme of *in vitro* percutaneous absorption experiments

Figure S1. Purification by flash chromatography of phloretin 4’-O-(6-O-octanoyl)-α-D-glucopyranoside **(1a).**

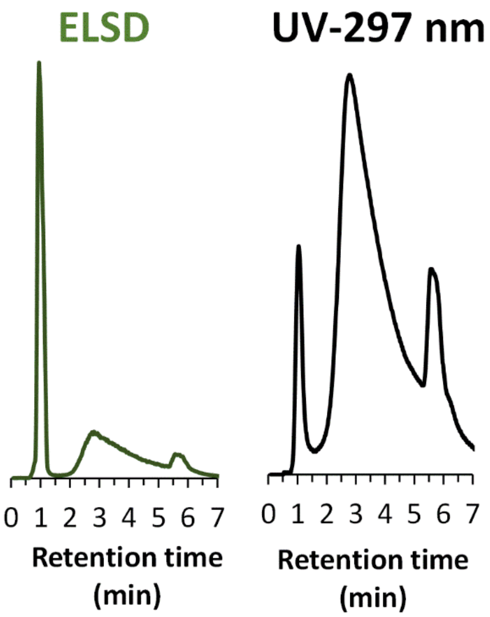
ELSD
UV-297 nm
0 1 2 3 4 5 6 7
Retention time (min)
0 1 2 3 4 5 6 7
Retention time (min)

Figure S2. ESI-MS of phloretin 4’-O-(6-O-octanoyl)-α-D-glucopyranoside **(1a).**

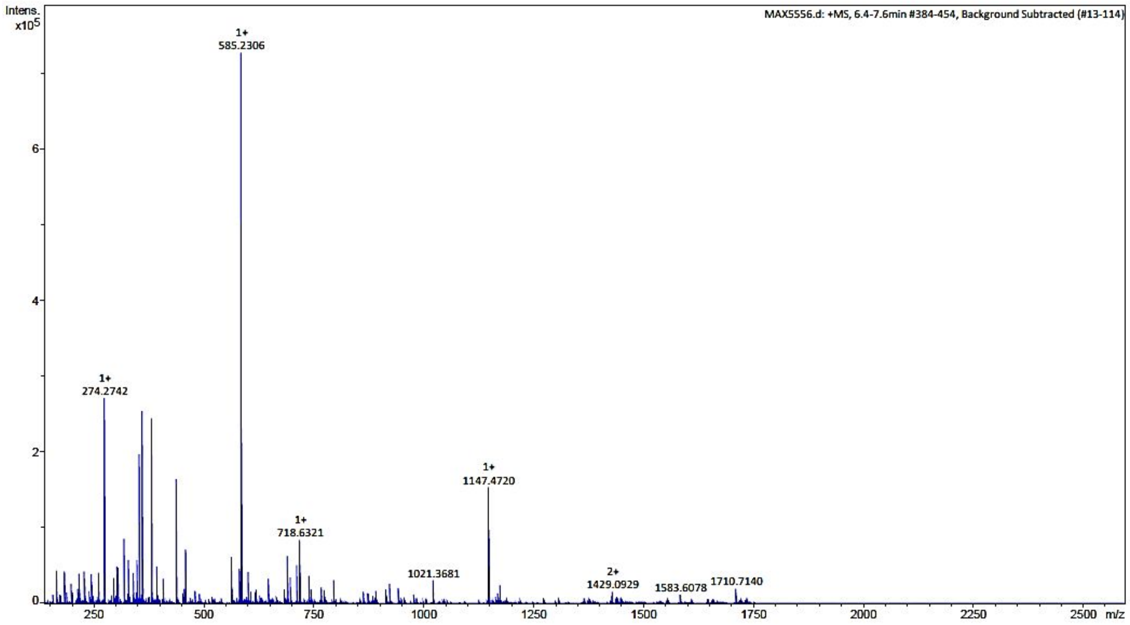

Figure S3. ESI-MS of phloretin 4’-O-(6-O-lauroyl)-α-D-glucopyranoside **(1b)**

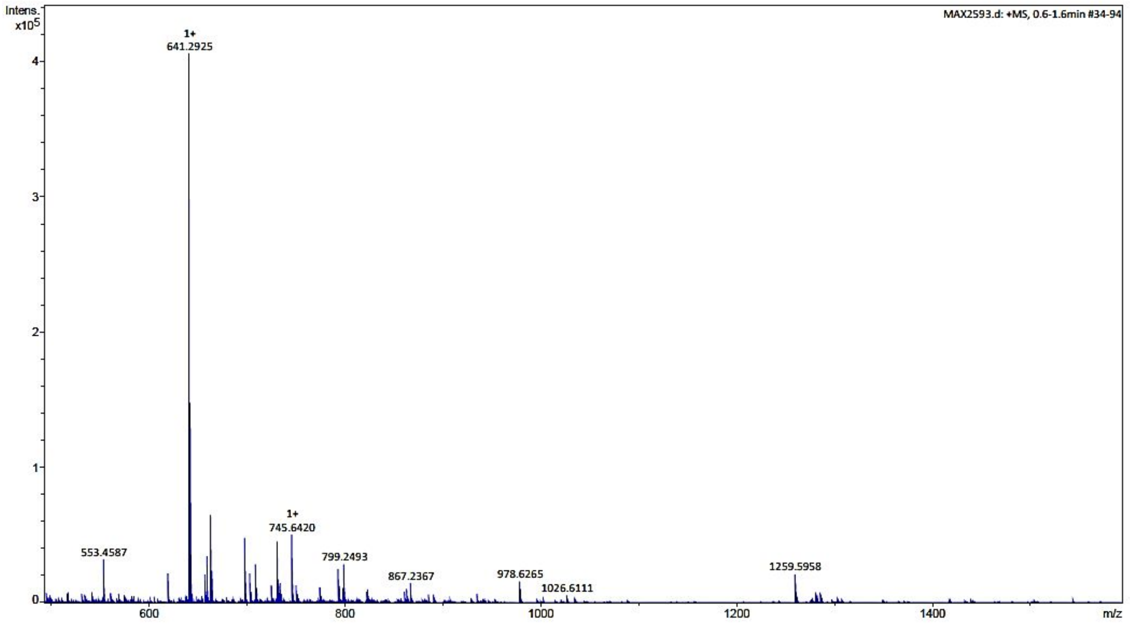

Figure S4. ESI-MS of phloretin 4'-O-(6-O-palmitoyl)-α-D-glucopyranoside **(1c)**

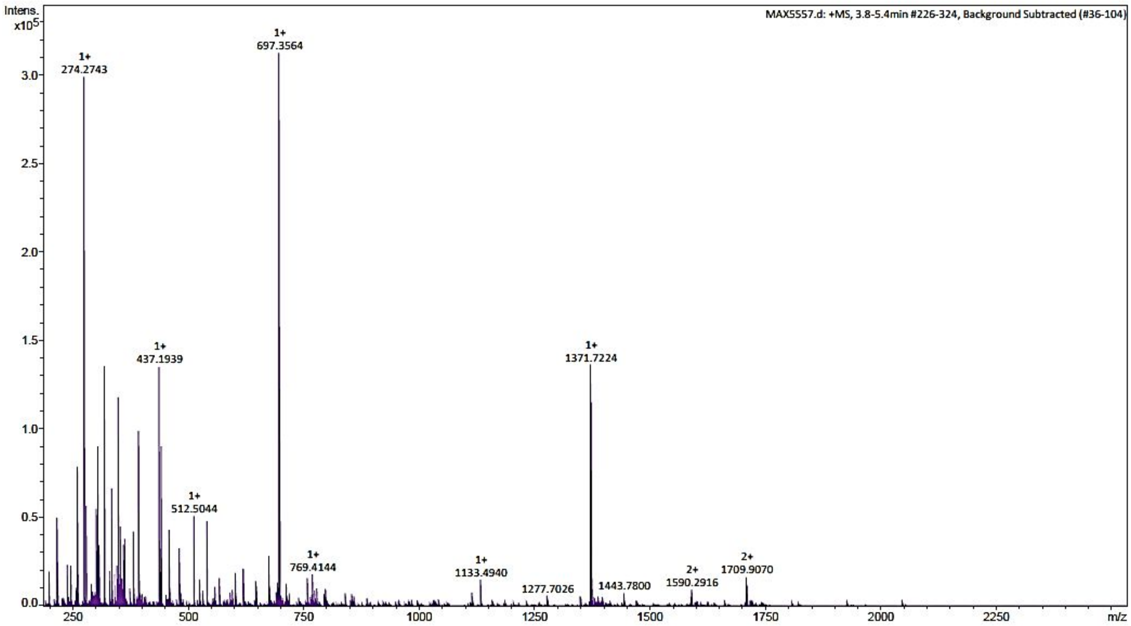

Figure S5. **(A)** 1H-NMR and **(B)** 13C-NMR of phloretin 4’-O-(6-O-octanoyl)-α-D-glucopyranoside **(1a)**

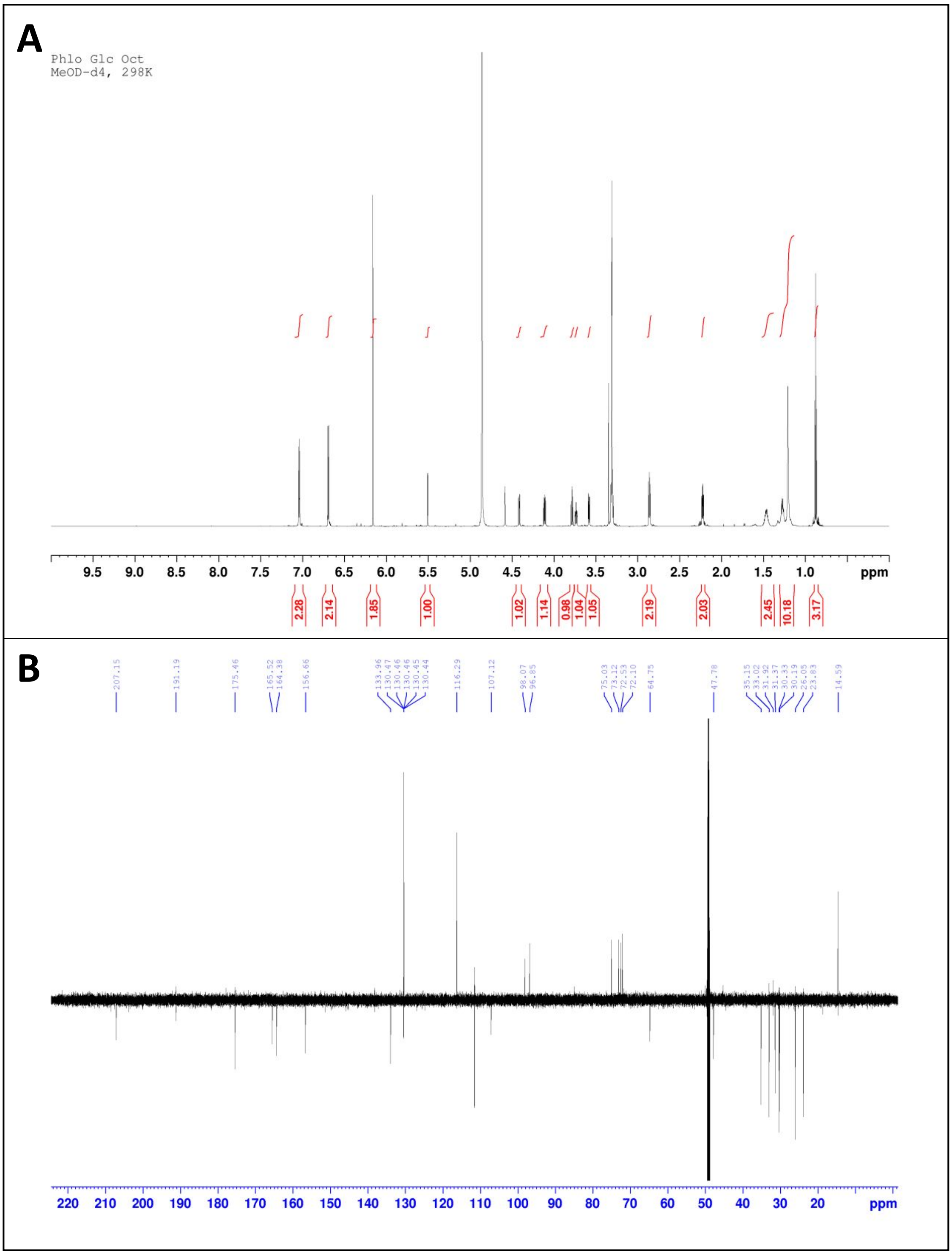

Figure S6. **(A)** HSQC and **(B)** HMBC of phloretin 4’-O-(6-O-octanoyl)-α-D-glucopyranoside **(1a)**

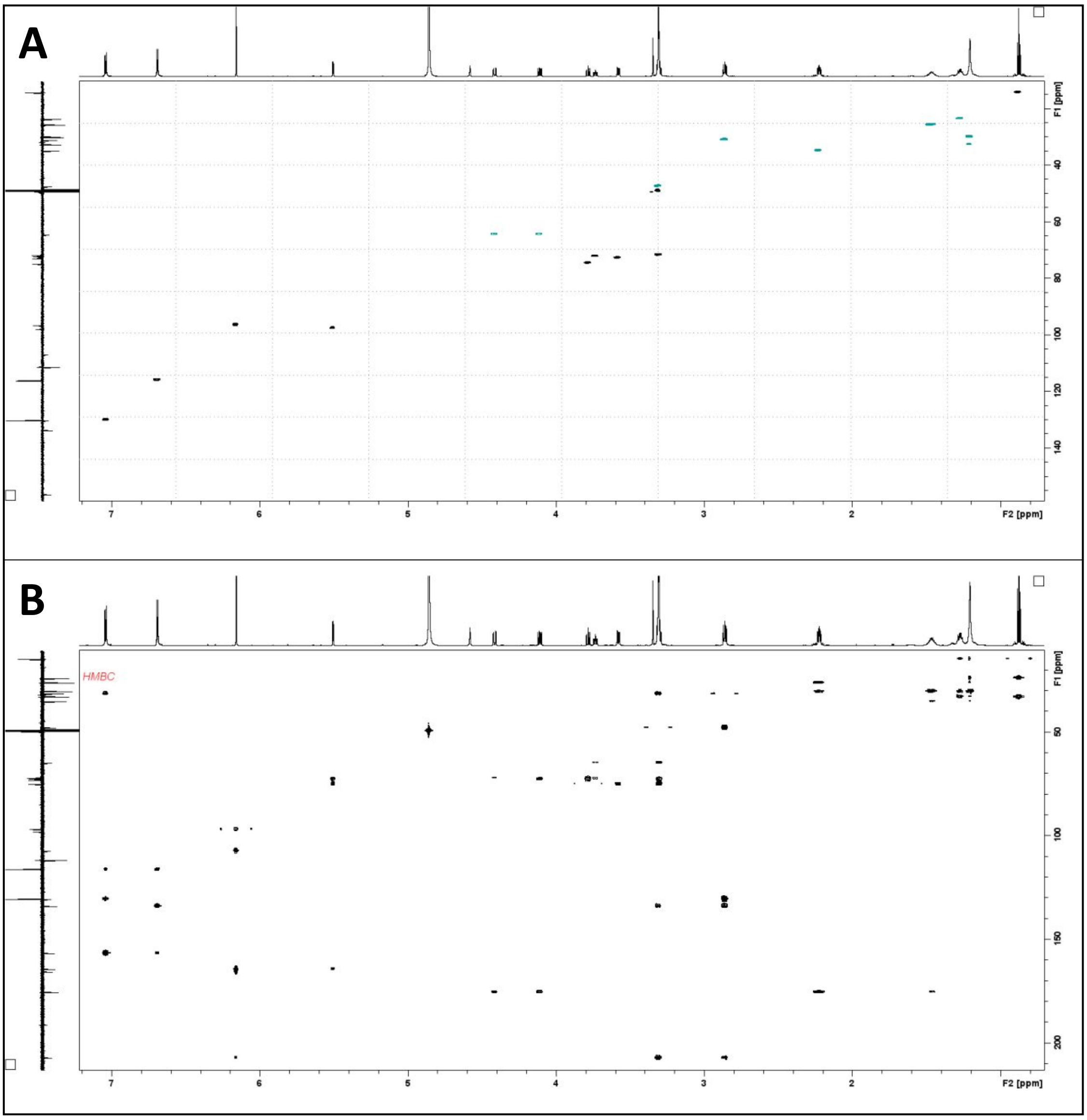

Figure S7. **(A)** 1H-NMR and **(B)** 13C-NMR of phloretin 4’-O-(6-O-lauroyl)-α-D-glucopyranoside **(1b)**.

A

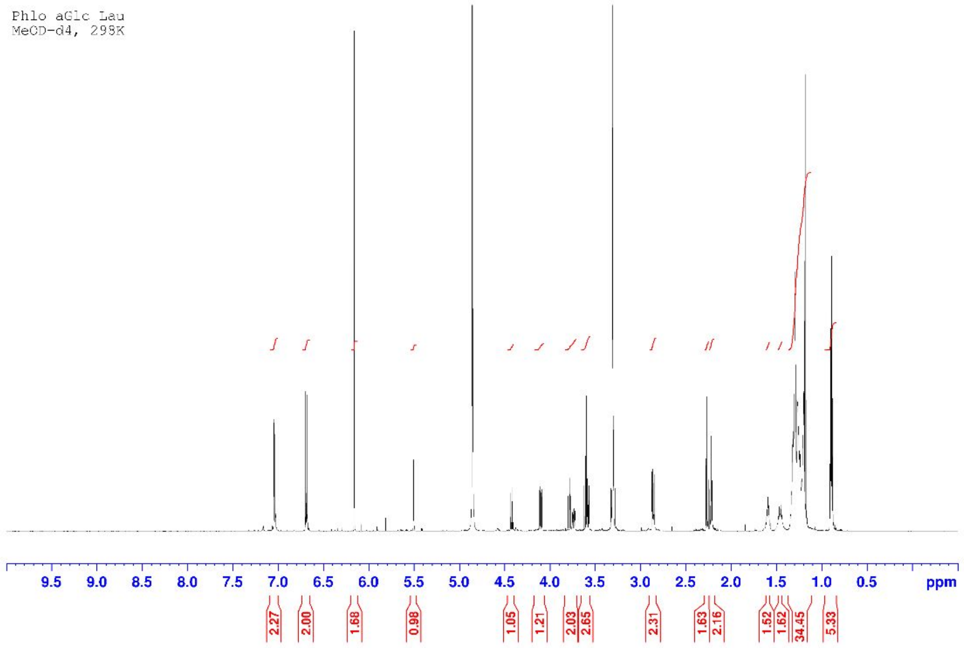

B

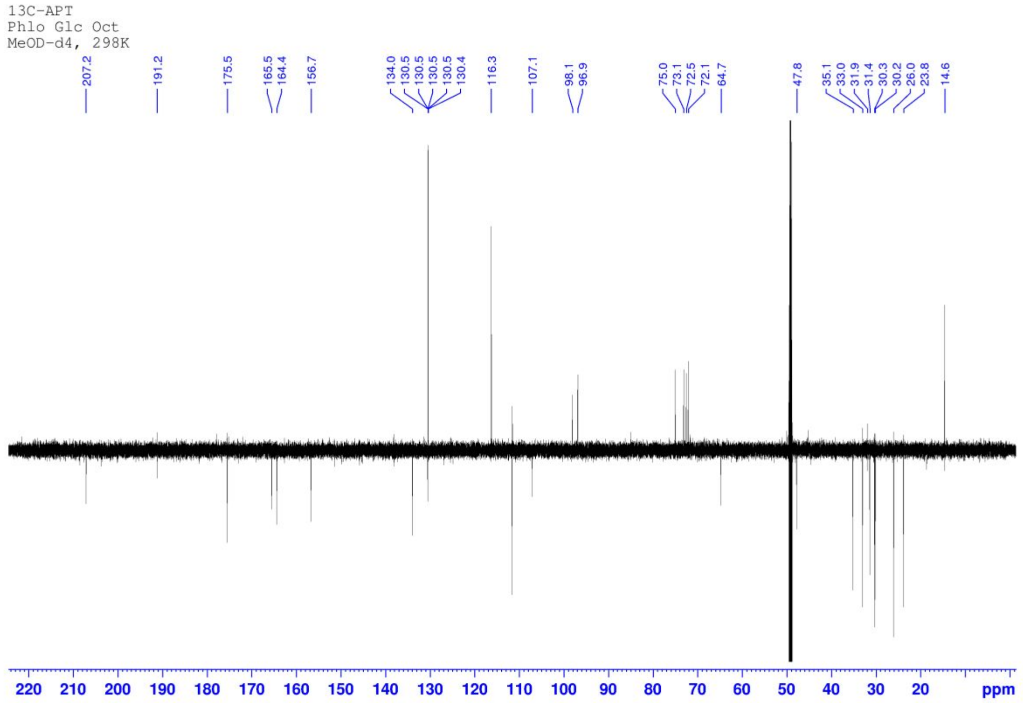

Figure S8. **(A)** HSQC and **(B)** HMBC of phloretin 4'-O-(6-O-lauroyl)-α-D-glucopyranoside **(1b)**.

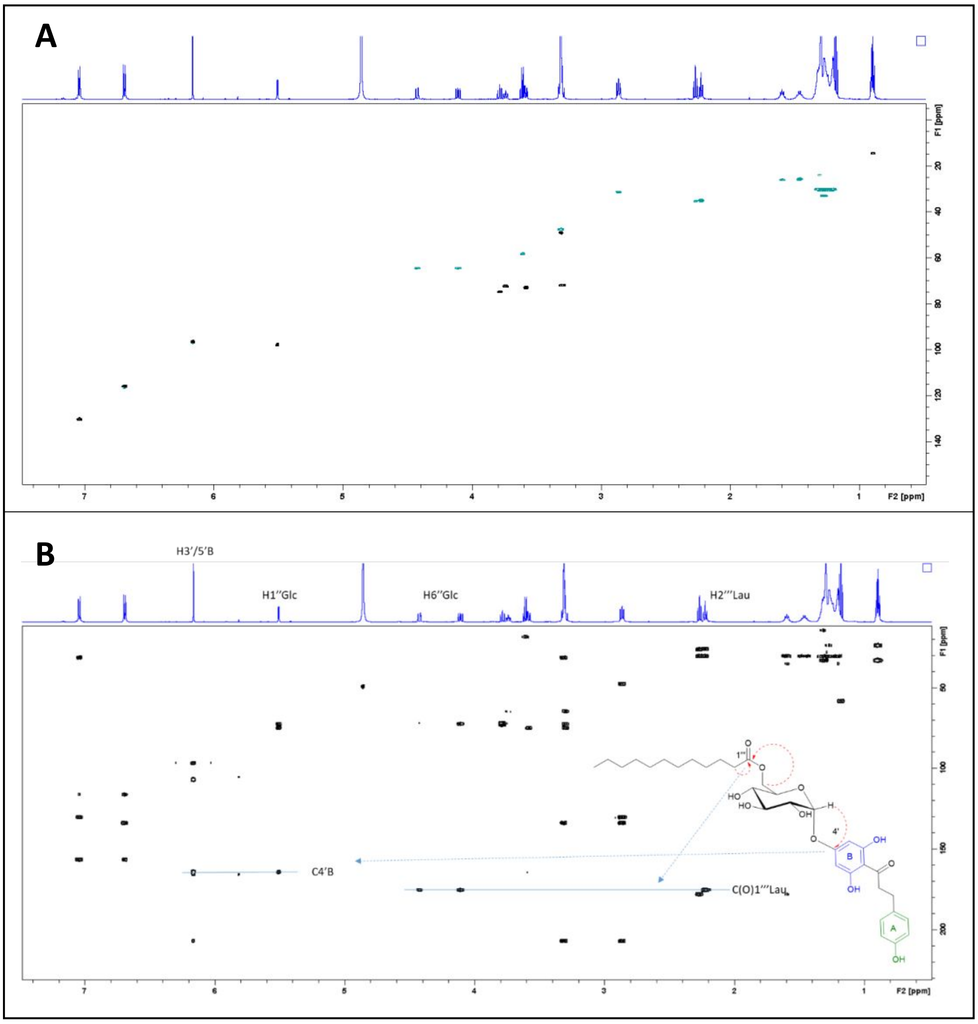

Figure S9. **(A)** 1H-NMR and **(B)** 13C-NMR of phloretin 4'-O-(6-O-palmitoyl)-α-D-glucopyranoside **(1c)**

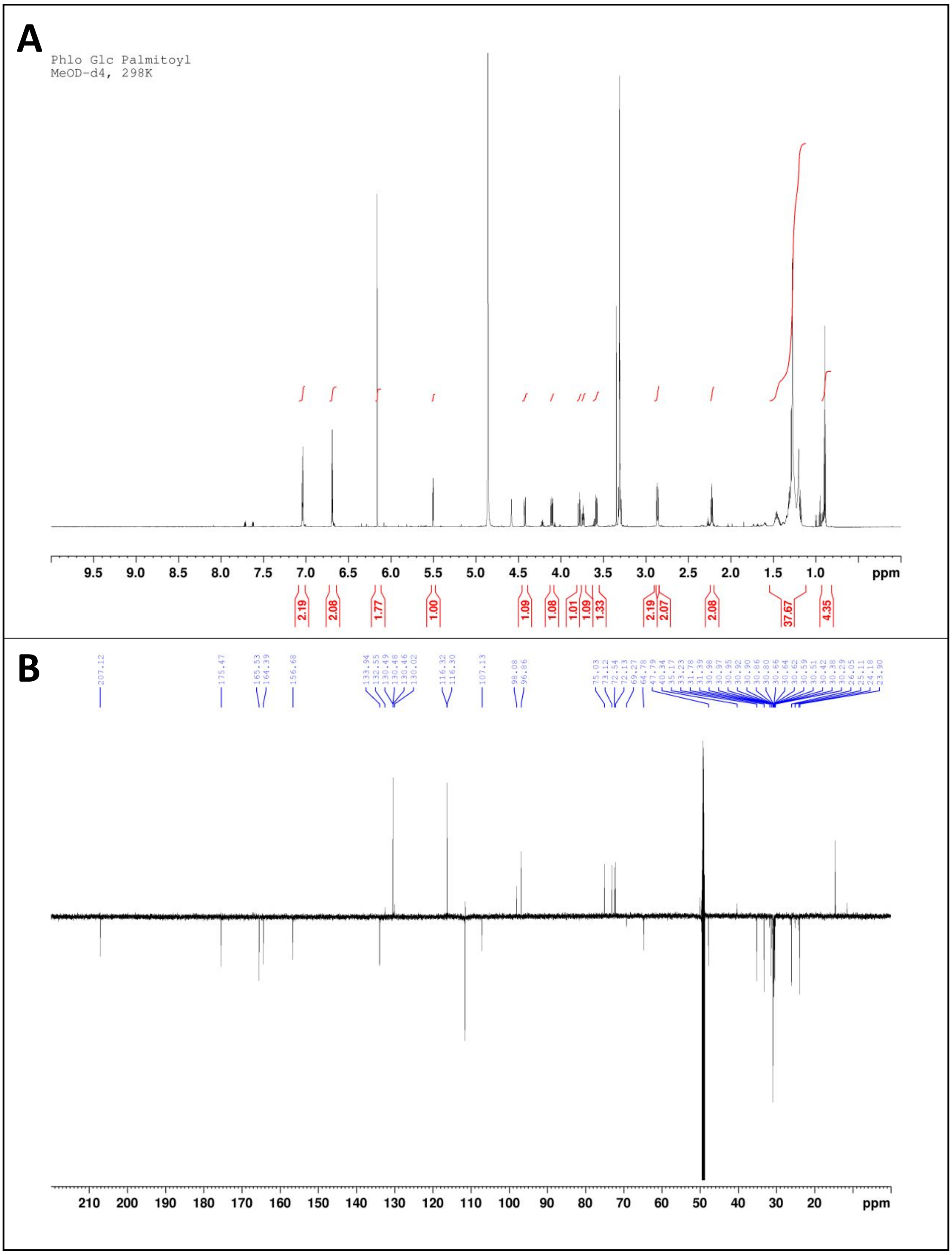

Figure S10. **(A)** HSQC and **(B)** HMBC of phloretin 4'-O-(6-O-palmitoyl)-α-D-glucopyranoside **(1c)**

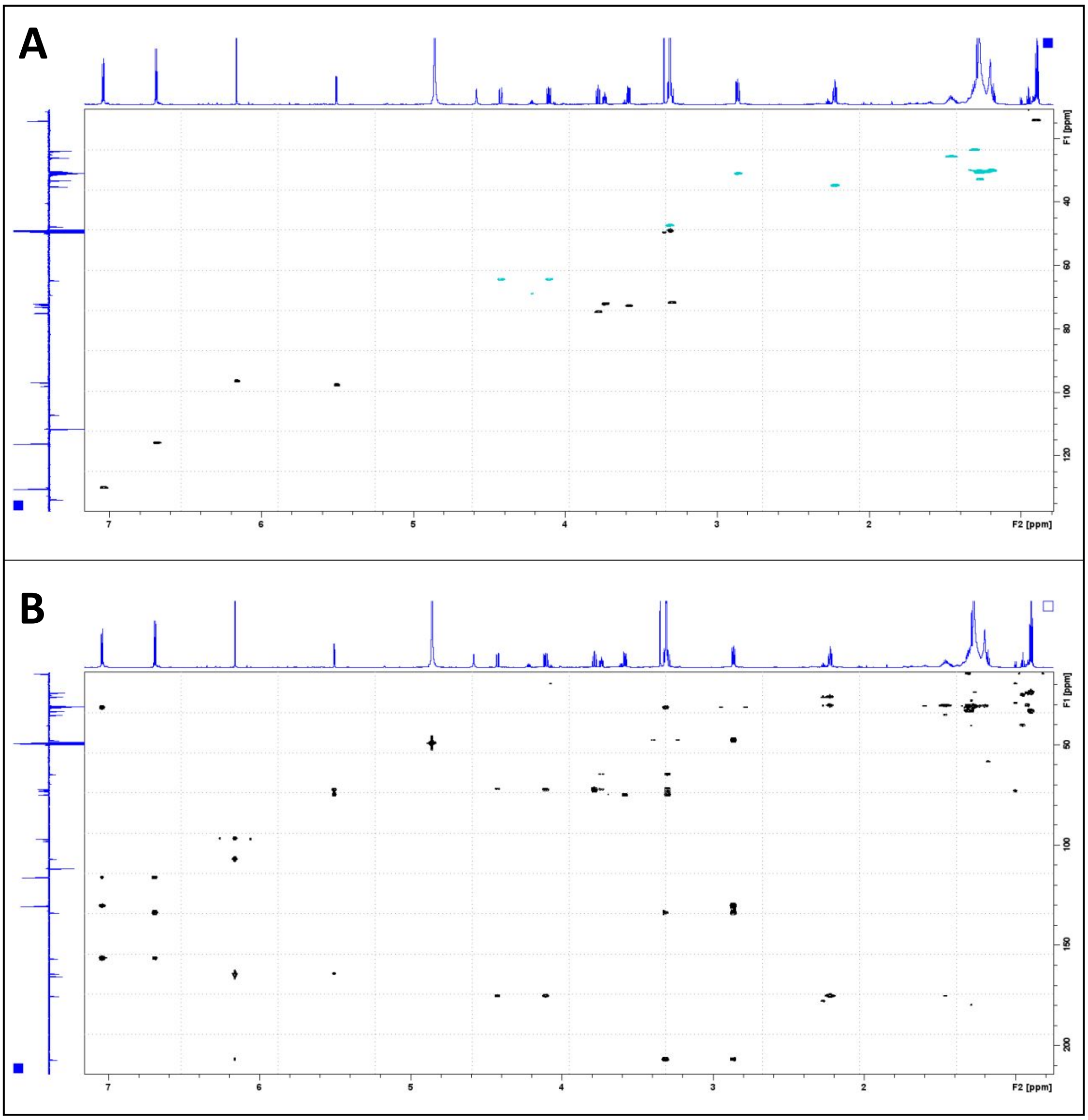

Figure S11. ^{1}H-NMR spectra of compounds **1a**, **1b** and **1c**. Some impurities are marked and identified as ethanol (*), methanol (◊) and free acid (○).

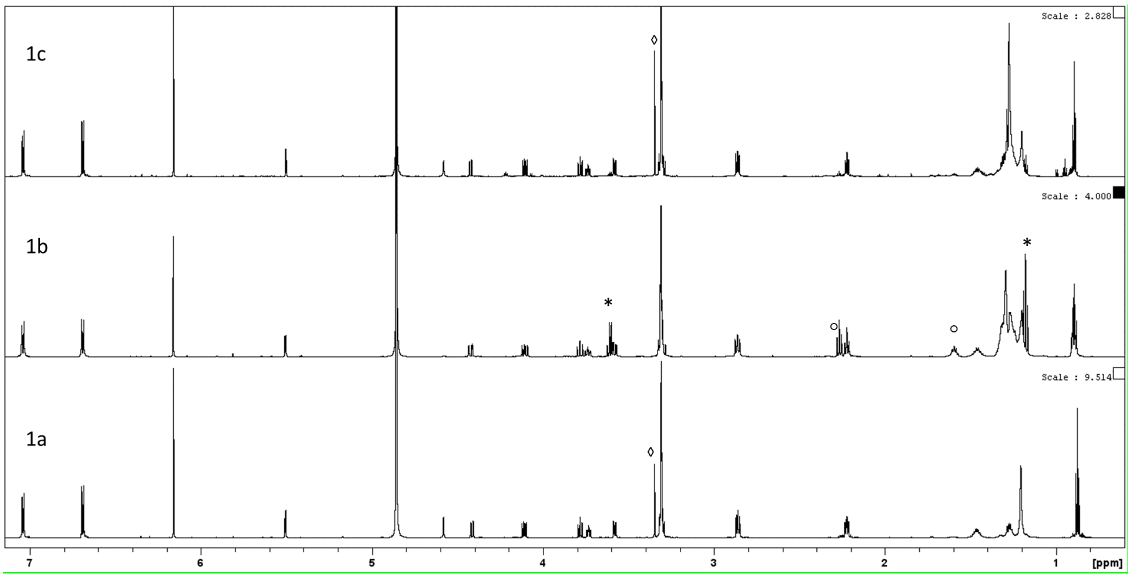

Figure S12. Stability of compounds **1a** and **1c** (approx. 1 mg/mL) in a 70:30 (v/v) mixture of ethanol and 100 mM buffer, at 37 ˚C. Buffers employed: sodium acetate (pH

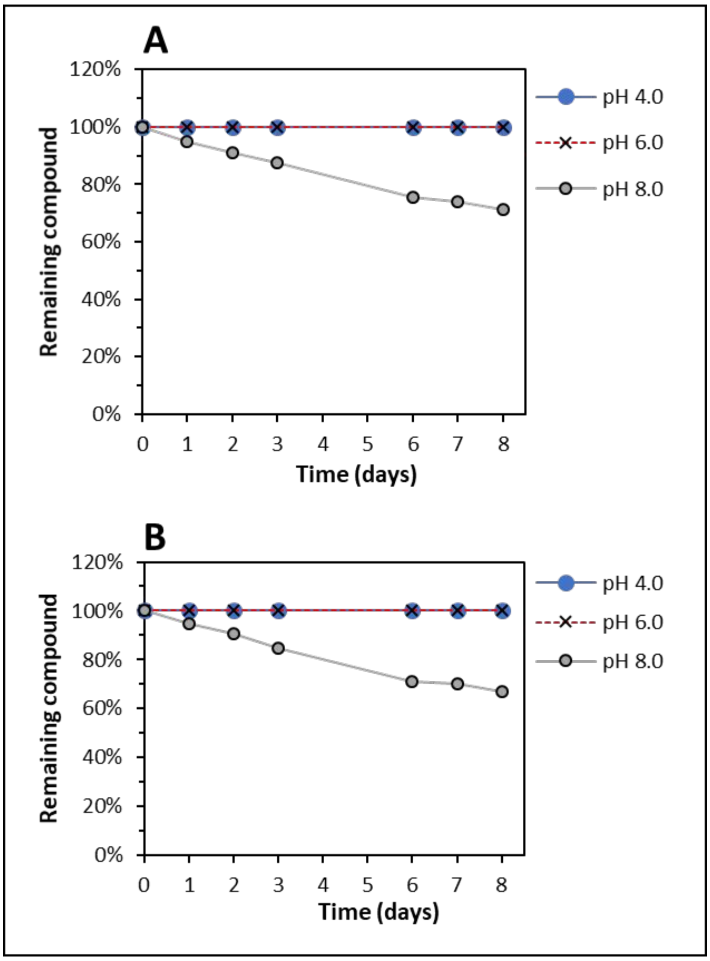

4.0), sodium phosphate (pH 6.0 and 8.0).

Figure S13. Scheme of *in vitro* percutaneous absorption experiments.

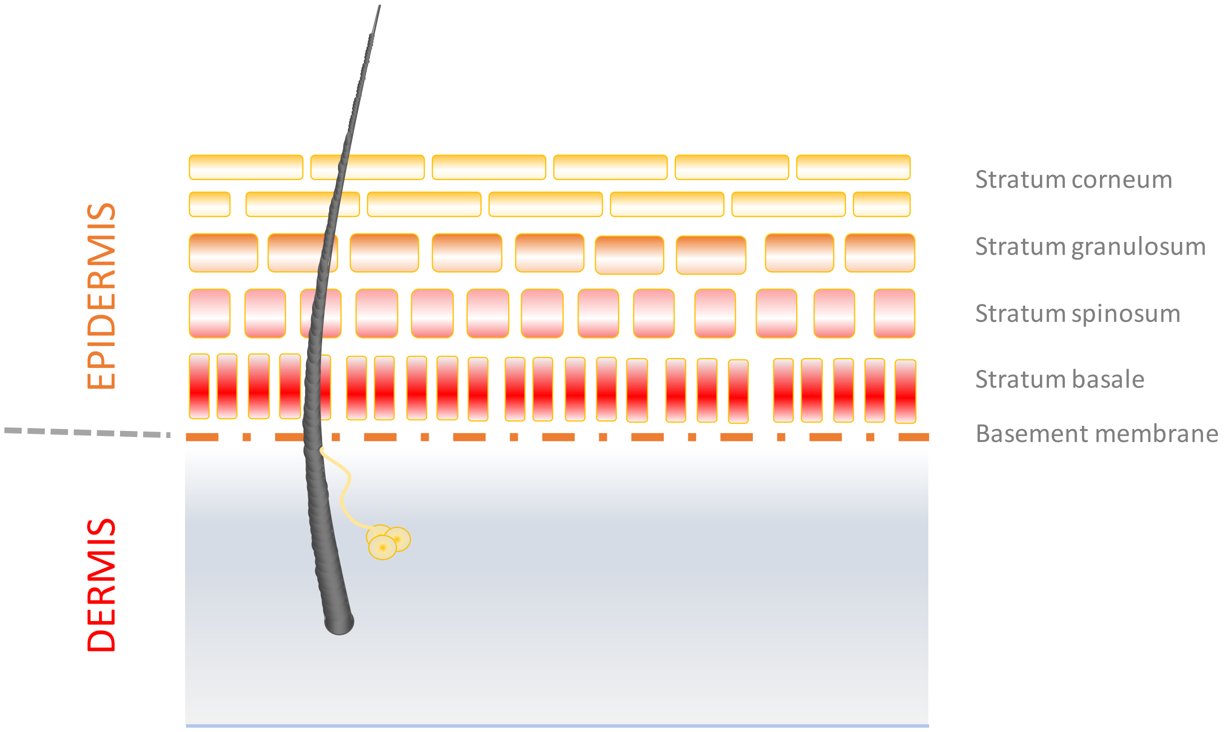
EPIDERMIS
DERMIS
Stratum corneum
Stratum granulosum
Stratum spinosum
Stratum basale
Basement membrane
